# Interaction Effect of Depression and Hypertension on Nephrotoxicity Among Persons Living With HIV: A Cross‐Sectional Study

**DOI:** 10.1155/ijne/8421994

**Published:** 2025-12-30

**Authors:** John Tetteh, Naana Agyeman, Swithin M. Swaray, Kwasi Torpey, Elijah Paintsil, Alfred Edwin Yawson, Duah Dwomoh

**Affiliations:** ^1^ Department of Community Health, University of Ghana Medical School, College of Health Sciences, University of Ghana, Accra, Ghana, ug.edu.gh; ^2^ Division of Musculoskeletal & Dermatological Sciences, Faculty of Biology, Medicine and Health, School of Biological Sciences, University of Manchester, Manchester, UK, manchester.ac.uk; ^3^ Department of Population, Family and Reproductive Health, School of Public Health, University of Ghana, Accra, Ghana, ug.edu.gh; ^4^ National Cardiothoracic Centre, Korle Bu Teaching Hospital, Accra, Ghana, kbth.gov.gh; ^5^ Department of Social and Behavioural Sciences, School of Public Health, University of Ghana, Accra, Ghana, ug.edu.gh; ^6^ Department of Pediatrics, Yale University School of Medicine, New Haven, Connecticut, USA, yale.edu; ^7^ Department of Biostatistics, School of Public Health, University of Ghana, Accra, Ghana, ug.edu.gh

**Keywords:** depression, HIV, hypertension, interaction, nephrotoxicity

## Abstract

**Background:**

The goal of antiretroviral therapy will not be achieved without addressing Human Immunodeficiency Virus (HIV) comorbidities, including depression, hypertension, and nephrotoxicity among people living with HIV (PLWH). This study was conducted to assess the interaction effect of depression and hypertension on nephrotoxicity among PLWH.

**Methods:**

The study employed a cross‐sectional study design. Data were collected from May to June 2022. The main outcome was nephrotoxicity, while depression and hypertension were considered as exposure factors. Confounders were identified through the directed acyclic graph and were controlled using the propensity score matching procedure. We estimated the causal association from the interactions by using weighted logistic regression.

**Results:**

The study involved 416 PLWH with ages ranging from 19 to 80 years (mean standard deviation was 49.32 ± 10.43 years). The majority (79.09%) of the participants involved were females. The prevalence of depression, hypertension, and nephrotoxicity was 21.87% (95% CI = 18.15–26.12), 36.30% (31.80–41.05), and 33.65% (29.26–38.35), respectively. Among participants with both depression and hypertension, analysis showed a substantial increase in the odds of nephrotoxicity. The proportion of the combined effect due to interaction was approximately 71% (63–77), and the excess risk due to interaction was positive (RERI = 1.87; 1.46–2.28). On the multiplicative scale, when both depression and hypertension are present, the risk of nephrotoxicity tripled (effect = 3.42; 2.70–4.14). Having depression raises the likelihood of nephrotoxicity by 55% (aOR = 1.55; 1.35–1.76) among PLWH with hypertension. Among PLWH with depression, the odds of nephrotoxicity increased by over twofold due to hypertension (aOR = 2.12; 1.83–2.42).

**Conclusion:**

The presence of both depression and hypertension raises the likelihood of nephrotoxicity much more than either condition alone. The findings revealed a synergistic effect, highlighting the need for integrated care that addresses both mental health and cardiovascular risks in HIV treatment.

## 1. Introduction

The Human Immunodeficiency Virus (HIV) continues to be a significant global health concern, affecting millions worldwide irrespective of age. Despite advancements in prevention and treatment, the global burden remains substantial. Over 79.3 million individuals have been infected since the onset of the pandemic, with approximately 37.7 million currently living with HIV [1, 2]. The African region bears the brunt, accounting for two‐thirds of global HIV cases, while over 346,000 people live with HIV in Ghana [3]. Advancements in antiretroviral therapy (ART) have led to a notable decrease in HIV‐associated mortality rates among people living with HIV (PLWH) in Ghana [4]. However, these regimens have been associated with toxicities and other comorbidities. Several studies have reported the emergence of ART‐associated cardiometabolic disorders [5–9]. These conditions are strongly associated with nephrotoxicity, particularly hypertension, diabetes, hyperlipidemia, hepatitis B, and/or hepatitis C co‐infection [5–9].

Meanwhile, the co‐existence of depression and hypertension is prevalent among PLWH [10], posing substantial health concerns. Depression, often undetected and untreated, can adversely impact immune function and hasten HIV disease progression [11]. Moreover, it is intricately linked with nephrotoxicity and renal complications [12–16]. Similarly, hypertension plays a role in the development of nephrotoxicity [17, 18].

However, the precise interplay between depression, hypertension, and nephrotoxicity among PLWH remains inadequately explored, particularly regarding how depression and hypertension interact to influence nephrotoxicity among PLWH. This gap is critical because the unique risks associated with the combined presence of these conditions are not fully understood, leaving healthcare providers without targeted strategies to mitigate these risks. Consequently, there is a pressing need to examine the interaction between depression and hypertension to know how they contribute to nephrotoxicity among PLWH. This study, therefore, focuses on assessing the combined effect of depression and hypertension on nephrotoxicity, shedding light on an often‐overlooked aspect of HIV care that is essential for optimizing long‐term health management in this population.

## 2. Methods

### 2.1. Study Site and Population

The study was conducted at the Korle‐Bu Teaching Hospital (KBTH) in Ghana. The study population was drawn from PLWH who receive care at the KBTH Infectious Disease Centre. Participants for the study were aged 18 years and above and were not referred from other facilities. The exclusion criteria were PLWH who were yet to start ART and were critically ill during the data collection period. Eligible participants who were pregnant and postpartum were also excluded from the study. Pregnant and postpartum women were excluded because physiological changes during these periods can transiently alter kidney function and blood pressure (BP), which could confound assessment of nephrotoxicity.

### 2.2. Research Design

A cross‐sectional study design was utilized to assess the interaction effect of depression and hypertension on nephrotoxicity among PLWH.

### 2.3. Sample Size and Sampling Procedure

The sample size calculation was based on interaction effect size. With a statistical power of 0.80, a two‐tailed significance level at (*α*) 5%, an assumed expected interaction effect of 0.30 on a log scale [19], and adjusting for a 15% nonresponse rate, the sample required for the study was 410. This was calculated using the formula below [20];

(1) n=4×Z1−α+Z1−β2d2,n=4×1.960.84+20.32,N=348.44.

We anticipated a dropout rate of 15% among the study population. To account for this, we adjusted our sample size estimate using the formula below.

(2) N=11−Q×n,N=110.15−×348.44,N≈410.

### 2.4. Primary Outcome

The main outcome of interest was nephrotoxicity. This was assessed by serum creatinine and used to estimate the glomerular filtration rate (eGFR) using the online calculator [21]. The presence of renal nephrotoxicity was defined as eGFR below 60 mL/min/1.73 m^2^. This form of the category was adopted from elsewhere [22]. Nephrotoxicity was defined based on a single eGFR measurement at enrollment, without prior or follow‐up assessments.

### 2.5. Exposure Factors

Depression and hypertension were the two main factors considered as exposures. The Patient Health Questionnaire (PHQ‐9) was used to assess depression [22]. The PHQ‐9 is made up of 9 standardized questions. Each describes a symptom and asks responders to assess it on a four‐point scale ranging from 0 (*not at all*) to 3 (*nearly every day*) based on how much it has troubled respondents in a fortnight. Total scores on the 9 items ranged from 0 to 27. A score of 10/27 was classified as depression based on previous literature [23]. The Cronbach’s alpha of the tool was 80%.

A digital BP monitor was used to measure hypertension taken on the upper arm over the brachial artery three times for all participants. It followed a standard protocol, which allowed the participant to rest quietly for at least 5 min before beginning the measurement, properly positioned the participant’s arm, and ensured that the cuff was positioned correctly around the upper arm [24–26]. The mean of the last two BP measurements was used to categorize hypertension status. Using the mean of the last two BP measurements helps to minimize variability and provides a more stable assessment of a patient’s current BP, improving accuracy in hypertension categorization. If BP is ≥ 140/90 mmHg, or if the patient is receiving antihypertensive medication, they are considered as having hypertension [27].

Four interactions were categorized in groups: (1) participants without depression and hypertension; (2) participants without depression but with hypertension; (3) participants with depression but without hypertension; and (4) participants with both depression and hypertension. These four responses were the main exposure of interest utilized to assess the interaction effect.

### 2.6. Independent Variable

To control factors that could affect the results, we collected various explanatory variables. These factors are presented in Appendix Table 1. Through a review of the literature and clinical relevance, we identified 26 possible factors that might influence the associations being studied [28–30].

### 2.7. Data Analysis

We started with a univariate descriptive analysis to summarize the study outcomes. Then, we used binary logistic regression to examine the factors linked to the outcomes and exposures. This approach helped us control important confounders, ensuring the results were accurate when analyzing interaction effects.

### 2.8. Controlling for Confounding Factors

To control for confounding factors, we used the directed acyclic graphs (DAGs) to visually represent relationships between outcome, exposure, and independently associated factors from the bivariate logistic regression, which helped identify potential confounders affecting the relationship between exposures and outcome. By mapping these relationships, DAGs allowed us to systematically determine which variables needed to be controlled to reduce bias, thereby enhancing the study’s validity and ensuring that observed interaction associations were not due to underlying confounders.

Two approaches were employed to handle confounders: (a) the propensity score matching (PSM) procedure and (b) sensitivity by adjusting for the confounders. We used the logistic regression analysis to estimate the propensity score for each subject based on observed covariates and then matched individuals with similar scores across exposure status. This process created a balanced sample that mirrored a randomized controlled trial by equating the distribution of confounders across exposure status. By matching individuals on their propensity scores, PSM reduced the effects of confounding, enabling a more accurate estimation of the causal association of the exposures on the outcome.

Logistic regression analysis was finally employed to assess the weighted interaction effect of depression and hypertension on nephrotoxicity. In evaluating the individual and joint effects of depression and hypertension on nephrotoxicity, both additive and multiplicative interactions (MIs) were reported [31]. Additive interaction was estimated considering relative excess risk due to interaction (RERI) and attributable proportion (AP) using the following formulas:

(3) RERI=aOR11−aOR10−aOR01+aOR00.

The domains; OR_11_, OR_10_, aOR_01_, and OR_00_ are the odds of nephrotoxicity for the interaction Group 4, Group 3, Group 2, and Group 1 (the comparison group), respectively.

(4) AP=RERIaOR11⁣.

Multiplicative interaction (MI) was assessed using the formula:

(5) MI=aOR11aOR10⁣∗aOR01.

In all the analyses, estimates were reported with the corresponding 95% confidence interval, and a *p*‐value < 0.05 was deemed significant.

## 3. Results

The study involved 416 PLWH, ages ranging from 19 to 80 years (mean ± SD = 49.32 ± 10.43 years). The majority (79.1%) of participants involved were females. The duration of HIV illness ranged from a month to 30 years. Approximately 86.1% of participants were taking tenofovir disoproxil fumarate, lamivudine (also known as Epivir), and dolutegravir (TDF + 3TC + DTG) ART combinations, with the majority (72.6%) being on the current combination within 2 years. Approximately 24.6% were obese, 32.3% were overweight, and 4.1% were underweight. Fruit and vegetable intake was 33.2% among the participants. About 21.4% of the participants sometimes add salt to their food at the table (Table 1).

**Table 1:** Sociodemographic characteristics among persons living with HIV.

Variable	Frequency	Percentage
Age group (in years)		
≤ 29	17	4.1
30–39	50	12.0
40–49	142	34.1
50–59	135	32.5
≥ 60	72	17.3
Sex		
Male	87	20.9
Female	329	79.1
Years of illness (*n* = 414)		
< 1	35	8.5
2–5	55	13.3
6–9	71	17.2
10+	253	61.1
Min–Max	A month‐30 years	
Median (IQR)	11 (8)	
Current ART combination (*n* = 416)		
TDF + DTG + 3TC	358	86.1
ABC + DTG + 3TC	16	3.9
TDF + EFV + 3TC	11	2.6
Other	31	7.5
Years on current ART (*n* = 412)		
≤ 1	77	18.7
< 2	299	72.6
3+	36	8.7
Min–Max	A month‐4	
Median (IQR)	2	0.7
BMI (*n* = 415)		
Underweight	17	4.1
Normal	162	39.0
Overweight	134	32.3
Obese	102	24.6
Fruit intake quintile (*n* = 416)		
Low	139	33.5
Middle	138	33.3
High	138	33.3
Vegetable intake quintile (*n* = 416)		
Low	139	33.4
Middle	139	33.4
High	138	33.2
Add salt at table (*n* = 416)		
Sometimes	89	21.4
Never	327	78.6
Food insecurity (*n* = 416)		
No	303	72.8
Yes	113	27.2

The prevalence of depression, hypertension, and nephrotoxicity was 21.87% (95% CI = 18.15–26.12), 36.30 (95% CI = 31.80–41.05), and 33.65 (95% CI = 29.26–38.35) (Appendix Table 2). As presented in Table 2, the prevalence of nephrotoxicity was higher (prevalence = 45.83%; 95% CI = 27.44–65.43) among patients who were depressed and had hypertension. This was followed by those with depression and hypertension free (35.82%; 25.27–47.74).

**Table 2:** Prevalence of nephrotoxicity by depression and hypertension interaction.

Model	Prevalence	95% CI
Multiplicative model		
−Depressed & −HPT (*n* = 198)	33.83	27.57 to 40.73
−Depressed & +HPT (*n* = 127)	29.92	22.58 to 38.46
+Depressed & −HPT (*n* = 67)	35.82	25.27 to 47.74
+Depressed & +HPT (*n* = 24)	45.83	27.44 to 65.43

*Note:* HPT = hypertension; *n* = number of participants for the related category.

Abbreviation: CI = confidence interval.

We observed a significant increase in hypertension and nephrotoxicity as age increases in women compared to younger groups (*p* < 0.001). In men, hypertension also increased significantly with age (*p* = 0.006), though nephrotoxicity remained relatively low across all groups (Appendix Table 6). These findings suggest that age‐related risk is more pronounced in women, consistent with possible postmenopausal influences.

### 3.1. Control for Confounding Factors Through the DAG

From the bivariate analysis, depression was associated with factors such as age, sex, asset quintile, years on ART, ART switching, BMI, fruit intake, vegetable intake, and adding salt at the table. Hypertension was linked to age, marital status, employment, number of children, comorbidities, BMI, fruit intake, and adding salt at the table. Nephrotoxicity was associated with age, sex, region, comorbidities, ART combination, HIV disclosure, and adding salt at the table (Appendix Tables 3–5).

**Figure 1:**
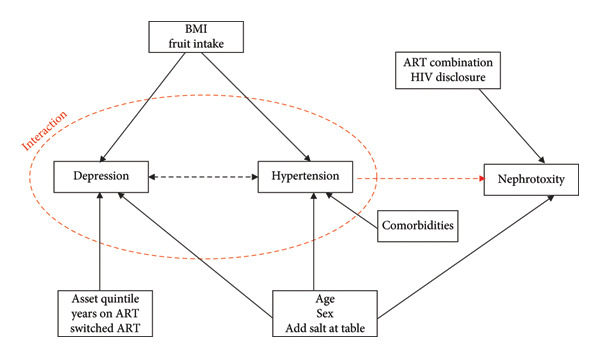
The directed acyclic graph showing the relationship between outcome, exposures, and confounding variables.

The DAG as presented in Figure 1 illustrates the causal relationships and common causes among depression and hypertension interaction and nephrotoxicity, as well as the associated factors influencing these conditions. The common causes for depression and hypertension interaction and nephrotoxicity were age, sex, and the addition of salt at the table.

**Figure 2:**
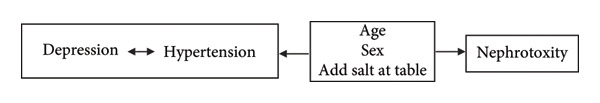
Possible pathway showing the relationships.

The possible pathway connecting this causal relationship is presented in Figure 2 below.

Because BMI and fruit intake were associated with depression and hypertension, these variables had no link to nephrotoxicity. Therefore, these variables were not controlled. Similarly, other factors with no link to nephrotoxicity or the exposures were not controlled. Not all variables collected in Appendix 1 were used in the PSM procedure. Variables included in the PSM were those identified as potential confounders through the DAG and bivariate analyses. Specifically, age, sex, and addition of salt at the table were included to control for confounding in the relationship between the depression × hypertension interaction and nephrotoxicity.

### 3.2. Interaction Effect of Depression and Hypertension on Nephrotoxicity Among Persons Living With HIV

Table 3 presents the interaction effect of hypertension and depression on nephrotoxicity among participants. Analysis showed that, for individuals with depression but without hypertension, the odds of nephrotoxicity increased by 3%; however, it was statistically not significant (aOR = 1.03; 95% CI = 0.96–1.10). For those with hypertension but without depression, the odds of nephrotoxicity counterintuitively reduced by 25% (aOR = 0.75; 0.71–0.80). Among participants with both depression and hypertension, analysis showed a substantial and statistically significant increase in the odds of nephrotoxicity (aOR = 2.06; 1.85–2.30). The proportion of the combined risk that is due to interaction was approximately 71% (63–77), and the excess risk due to interaction slightly increased by 87% (RERI = 1.87; 1.46–2.28). On a multiplicative scale, when both depression and hypertension are present, the risk of nephrotoxicity increased by over threefold (effect = 3.42; 2.70–4.14). This implies that the presence of both conditions triples the risk, highlighting a combined effect where the two conditions together significantly raise the odds of nephrotoxicity.

**Table 3:** Weighted interaction effect of hypertension and depression on nephrotoxicity among people living with HIV.

aOR depression by hypertension	No depression	Depression	aOR due to depression
No hypertension	1 (Reference)	1.03 [0.96 to 1.10]	1.03 [0.96 to 1.10]
Hypertension	0.75 [0.71 to 0.80]^∗∗∗^	2.06 [1.85 to 2.30]^∗∗∗^	1.55 [1.35 to 1.76]^∗∗∗^
aOR due to hypertension	0.75 [0.71 to 0.80]^∗∗∗^	2.12 [1.83 to 2.42]^∗∗∗^	1.60 [1.34 to 1.87]^∗∗∗^

^∗∗∗^ *p* < 0.001.

When examining the influence of each condition in the presence of the other, the analysis showed that among individuals with hypertension, having depression raises the likelihood of nephrotoxicity by 55% (aOR = 1.55; 1.35–1.76). Conversely, among those with depression, the odds of nephrotoxicity increased by over twofold due to hypertension (aOR = 2.12; 1.83–2.42). Thus, PLWH who had hypertension and depression increased the odds of nephrotoxicity by 60% (aOR = 1.60; 1.34–1.87). The same evidence exists when depressed PLWH had hypertension.

### 3.3. Predictions

**Figure 3:**
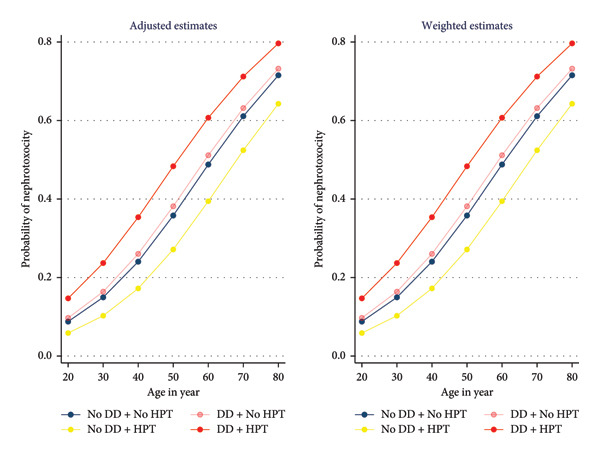
Probability of nephrotoxicity according to depression and hypertension interaction.

The probability of nephrotoxicity across different age groups, considering the interaction between depression and hypertension, is presented in Figure 3 below. The adjusted estimates showed results after accounting for the confounders and controlling for PSM estimates. Both graphs indicated that as age increases, the probability of nephrotoxicity also increases. Individuals with both depression and hypertension consistently show a higher risk across all ages. Those without depression and hypertension exhibited the lowest probability of nephrotoxicity across age groups. The combination of depression and hypertension was associated with a higher likelihood of nephrotoxicity, especially as age progresses.

**Figure 4:**
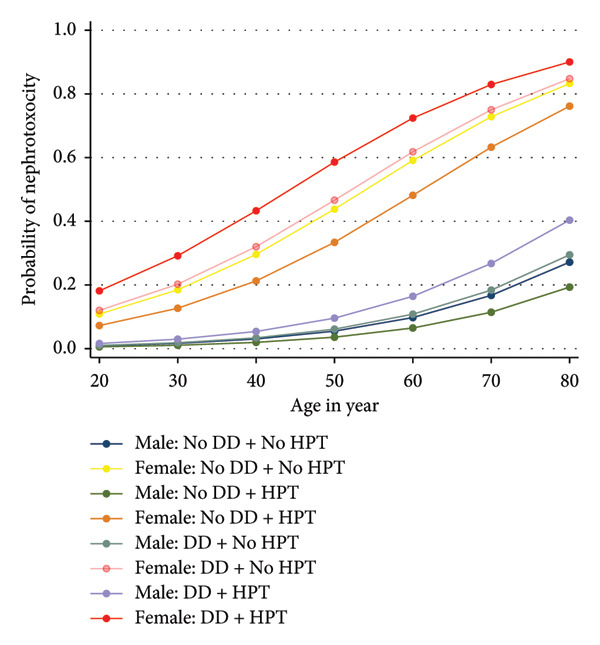
Probability of nephrotoxicity according to depression and hypertension interaction by sex differential. Estimates from weighted logistic regression analysis.

Females with both depression and hypertension have the highest probability of nephrotoxicity at all ages. Males with both conditions also show an elevated risk, although significantly lower than their female counterparts (Figure 4).

## 4. Discussion

The findings of this study revealed a significant interaction between depression and hypertension that exacerbates the risk of nephrotoxicity among PLWH. The combination of both conditions increases the risk of developing nephrotoxicity, indicating a compounded effect that surpasses the risk posed by either condition alone. This interaction suggests that when depression and hypertension coexist, they substantially heighten vulnerability to kidney disease. Interestingly, while hypertension alone counterintuitively was associated with reduced odds, depression alone slightly increased the odds, though not significantly. These results point to a complex interplay where the concurrent presence of both conditions amplifies nephrotoxicity risk more than either does individually.

The observed interaction effect between depression and hypertension on nephrotoxicity can be explained by the fact that depression and hypertension likely share common pathways that contribute to kidney damage [32]. Both conditions are known to be stress‐reactive, potentially triggering or exacerbating nephrotoxicity through mechanisms linked to prolonged inflammatory and neuroendocrine responses [33–35]. Chronic stress from depression and the physiological strain from hypertension may jointly increase oxidative stress and inflammatory cytokine production, both of which are recognized contributors to kidney damage [34, 36–38]. Additionally, the interaction between these stress‐reactive diseases can lead to dysregulation of the hypothalamic–pituitary–adrenal (HPA) axis, further aggravating renal impairment [39]. However, the exact mechanisms linking depression, hypertension, and nephrotoxicity remain uncertain and warrant further investigation to clarify these complex pathways and to inform targeted interventions for PLWH who suffer from these comorbid conditions.

The seemingly low odds ratio for hypertension alone should be interpreted with caution. This finding may reflect the influence of treatment effects, variability in BP control, or differences in disease severity among participants. Additionally, the cross‐sectional nature of the study limits our ability to infer causality. Future longitudinal investigations incorporating repeated BP assessments, treatment history, and care‐related factors are warranted to further elucidate these relationships.

Additionally, age and gender also influence nephrotoxicity risk in PLWH with depression and hypertension. With increasing age, the likelihood of nephrotoxicity rises, with the combination of both conditions maintaining the highest risk across age groups. This trend is particularly pronounced among females, who consistently exhibit a greater risk of nephrotoxicity than their male counterparts when both depression and hypertension are present. The predominance of females (approximately 80%) in our study sample reflects the broader epidemiological pattern of HIV infection in Ghana, where women continue to bear a disproportionate burden of the disease [40]. Thus, the observed sex distribution is representative of the national HIV demographic profile and enhances the relevance of our findings. These findings underscore the need for targeted interventions that integrate mental health and cardiovascular care into HIV treatment, especially for older adults and females, to reduce nephrotoxicity risks and enhance overall health outcomes in this population. Although we did not collect reproductive or menopausal status, stratification by age in women showed higher prevalence of hypertension and nephrotoxicity as women’s age increases, consistent with literature on postmenopausal cardiovascular–renal risk [41]. However, the cross‐sectional nature of the study and lack of hormonal data preclude definitive conclusions, and future studies should investigate reproductive factors in relation to nephrotoxicity.

ART can disrupt hormonal balance, influencing both mental health and cardiometabolic outcomes. Some regimens cause metabolic changes that may interact with sex hormones. In women, loss of estrogen with age removes a key protective factor [42], and ART‐related stressors may heighten vascular and kidney risk. Hormonal shifts are also linked to depression [43], suggesting shared pathways between mental and kidney health. Although we did not measure hormones, these mechanisms may help explain the higher nephrotoxicity seen in older women. Future studies should include hormonal and reproductive markers to clarify these links. The study showed high prevalence rates of hypertension (36.30%), nephrotoxicity (33.65%), and depression (21.87%), indicating a substantial comorbidity burden that could affect their quality of life. The depression rate aligns closely with regional estimates, falling between the 17.9% reported in Côte d’Ivoire and Senegal [44] and the 28.6% found in Cape Coast, Ghana [42]. Hypertension prevalence matches findings from Brazil and Kenya [45, 46] and correlates with previous results in Ghana [47], though it exceeds lower rates observed elsewhere in Africa, like Kenya and Cameroon [48–50]. Nephrotoxicity rates here are lower than those in the Philippines (51.38%) [51] but significantly higher than in France (3.7%) [52] and Ethiopia (16.1%) [53], suggesting geographic variations. These findings highlight the need for targeted health interventions to address these prevalent conditions in PLWH.

### 4.1. Strengths and Limitations

A notable strength of the study is the adoption of robust analysis techniques involving both statistical and epidemiological approaches. These analyses addressed the research objectives. The novelty of the study also serves as a key strength, evidencing the impact of both depression and hypertension on nephrotoxicity. This evidence is new globally and serves as a platform for further studies. In addition, both public health and clinical outcomes were assessed in this single study, and the process adopted to estimate these outcomes was robust and standardized.

This study has several limitations that should be considered when interpreting the findings. First, the cross‐sectional design precludes causal inferences. However, to minimize potential bias and strengthen the validity of the observed associations, a causal inference framework guided by DAGs was rigorously applied in the study design and analysis. Another key limitation of this study is the potential for recall bias, which could arise from participants’ reliance on memory to report past medical conditions, symptoms, or treatment experiences. Some variables, like depression, were assessed over the past 2 weeks. The researchers believed the recall periodmay be associated with some form of bias. However, these periods are standard ways of measuring the respective outcomes, which have been adopted elsewhere [22]. In addition, BP was measured cross‐sectionally, which increases the potential for misclassification due to white‐coat or masked hypertension. Additionally, many participants classified as hypertensive were under antihypertensive treatment; thus, adequate BP control may have attenuated the observed association with nephrotoxicity. However, we lacked detailed data on antihypertensive drug classes, treatment duration, and prior BP control, which could have influenced renal outcomes. We acknowledge that evaluating nephrotoxicity solely through eGFR provides a limited assessment compared to current KDIGO guidelines [54], which also recommend the use of proteinuria and microalbuminuria. However, due to resource and logistical constraints, additional biomarkers were not available in this study. Future studies should incorporate additional renal biomarkers to provide a more comprehensive evaluation of nephrotoxicity. Furthermore, residual confounding from these unmeasured factors, as well as potential selection or survivor bias, cannot be ruled out.

## 5. Conclusion

Overall, the findings showed that the presence of both depression and hypertension substantially increases the likelihood of nephrotoxicity compared with either condition alone. Age and gender further modulate this risk, with older adults and females particularly susceptible, underscoring the need for tailored and proactive management in these groups. These results highlight the importance of integrated care models that combine HIV treatment with cardiovascular and mental health services. Evidence from hypertension programs has shown that consistent BP control can reduce the risk of kidney injury [55], while mental health interventions, including counseling and treatment for depression, have been associated with better adherence to ART and improved physical outcomes among PLWH [56]. Strengthening such interventions within HIV care could help reduce nephrotoxicity and improve overall health outcomes. Future longitudinal studies are warranted to confirm these associations and to evaluate the effectiveness of integrated mental health and hypertension management in reducing renal complications in PLWH.

## Ethics Statement

This study was approved by the Korle‐Bu Teaching Hospital Scientific and Technical Committee (Ethical Approval No. KBTH‐STC/IRB/00025/2022). Permission to conduct the study was obtained from the Fevers Unit, Korle‐Bu Teaching Hospital, prior to data collection. The study was conducted in accordance with relevant institutional and national guidelines and regulations and with the principles of the Declaration of Helsinki.

## Consent

Written informed consent was obtained from all participants before recruitment. For participants who were unable to read, the consent form was read and explained by a trained research assistant, and verbal consent was obtained in the presence of an independent witness; the witness attested to the participant’s voluntary agreement and signed the consent form.

Participants gave explicit consent for the use of anonymized data and aggregated results in publications. No identifiable personal information that could identify participants is included in this manuscript.

## Disclosure

The content is solely the responsibility of the authors and does not necessarily represent the official views of the National Institutes of Health.

## Conflicts of Interest

The authors declare no conflicts of interest.

## Author Contributions

Conceptualization: John Tetteh and Duah Dwomoh.

Methodology: John Tetteh and Duah Dwomoh.

Investigation (data collection): John Tetteh and Swithin M. Swaray.

Formal analysis: John Tetteh.

Writing–original draft: John Tetteh, Naana Agyeman, and Swithin M. Swaray.

Writing–review and editing: John Tetteh, Naana Agyeman, Swithin M. Swaray, Kwasi Torpey, Elijah Paintsil, Alfred Edwin Yawson, and Duah Dwomoh.

## Funding

The research reported in this publication was supported by the Fogarty International Center and the National Institute of Alcohol Abuse and Alcoholism of the National Institutes of Health under Award Number D43 TW011526.

## Supporting Information

Appendix Table 1. Study variables and definitions: This appendix outlines all variables used in the analysis and provides their definitions, measurements, and scale of measurements. The variables include sociodemographic characteristics, HIV‐related factors, and nutritional indicators.

Appendix Table 2. Descriptive analysis and prevalence of outcomes: This appendix presents descriptive statistics and the prevalence of depression, hypertension, and nephrotoxicity by sociodemographic characteristics among persons living with HIV.

Appendix Tables 3–5. Factors associated with health outcomes: These appendices show the associations between sociodemographic, HIV‐related, anthropometric, and nutritional factors and the outcomes of depression, hypertension, and nephrotoxicity among persons living with HIV.

Appendix Table 6. Distribution by sex and age: This appendix presents the distribution of depression, hypertension, and nephrotoxicity stratified by sex and age groups.

## Supporting information


**Supporting Information** Additional supporting information can be found online in the Supporting Information section.

## Data Availability

The de‐identified individual participant data that support the findings of this study are available from the corresponding author upon reasonable request and subject to institutional data‐sharing agreements and ethical approvals to protect participant confidentiality. Study documents (e.g., study protocol, data dictionary, and statistical analysis) are available upon reasonable request.
