# Supplementary material for: Interaction Effect of Depression and Hypertension on Nephrotoxicity Among Persons Living With HIV: A Cross‐Sectional Study
Source: Int J Nephrol. 2025 Dec 30;2025:8421994. doi: 10.1155/ijne/8421994 (PMC12754264; doi:10.1155/ijne/8421994)
Supplement: Supplementary file 1 — Supporting Information Additional supporting information can be found online in the Supporting Information section. [file IJNE-2025-8421994-s001.docx]

**Appendix Table 1: Study variable and definitions**

| Age | Independent variable | Age of the participant | Raw counts as a continuous measure and categorical base on the nature of the data | Discrete Categorical |
| --- | --- | --- | --- | --- |
| Sex | Independent variable | Sex differential of participant | Male or Female | Binary |
| Education | Independent variable | The educational level of the participant | None, primary, secondary/MSLC, Tertiary | Categorical |
| Marital status | Independent variable | The marital status of the participant | Never married, married, separated/divorced | Categorical |
| Employment | Independent variable | Whether the participant is currently working or not | Yes or NO | Binary |
| Religion | Independent variable | Religious affiliation of the participant | None, Christian, Islam, Traditional/other | Categorical |
| Asset quintile | Independent variable | Participants wealth status | Low, middle, and high | Ordinal |
| Region of residence | Independent variable | Self-reported place of residence of the participant | Rural or Urban | Binary |
| Has valid NHIS | Independent variable | The participant having access to valid NHIS | Yes or No | Binary |
| Social support | Independent variable | Participants receiving support for treatment | Yes or No | Binary |
| Has sexual partner | Independent variable | The participant having a sexual partner to valid NHIS | Yes or No | Binary |
| Use of herbal medicine | Independent variable | Whether the participant currently using any herbal medicine for the treatment | Yes or No | Binary |
| Co-morbid condition | Independent variable | Participant has any comorbidity condition | Yes or No | Binary |
| Years of illness | Independent variable | Years in which the participant has lived with HIV | Raw counts as a continuous measure and categorical base on the nature of the data | Discrete  Categorical |
| Current ART combination | Independent variable | The type of ART combination participant is taking | TDF+DTG+3TC  ABC+DTG+3TC  TDF+EFV+3TC  Other | Categorical |

*Continued*

**Appendix Table 1 Cont.: Study variables and definitions**

| Years of current ART | Independent variable | Number of years the participant has been on ART | Raw counts as a continuous measure and categorical base on the nature of the data | Discrete  Categorical |
| --- | --- | --- | --- | --- |
| Any past ART | Independent variable | Whether the participant switched the ART regimen | Yes or No | Binary |
| Currently taking Septrin | Independent variable | Participant currently taking Septrin for protection | Yes or No | Binary |
| Disclosure of HIV | Independent variable | Whether the participant has disclosed HIV illness to anybody | None, Family members, Partners, and Freinds+Others | Categorical |
| BMI | Independent variable | Participant Body Mass Index | Underweight, normal, overweight, and obesity |  |
| Fruit intake | Independent variable | The frequency of fruit intake | Low, Middle, and High | Ordinal |
| Vegetable intake | Independent variable | The frequency of fruit intake | Low, Middle, High | Ordinal |
| Add salt to the table | Independent variable | A participant added salt to the table when eating | Yes or No | Binary |
| Food insecurity | Independent variable | The participant having access to less food to eat over the past 12 months | Yes or No | Binary |

**Appendix Table 2: Descriptive analysis and prevalence of depression, hypertension, and nephrotoxicity by sociodemographic characteristics among Persons Living with HIV**

| **Variable** | **Frequency** | **Depression** | **Hypertension** | **Nephrotoxicity** |
| --- | --- | --- | --- | --- |
|  | **N (%)** | **Proportion [95%CI]** | **Proportion [95%CI]** | **Proportion [95%CI]** |
| **Overall prevalence** | **N = 416** | 21.87 [18.15-26.12] | 36.30 [31.80-41.05] | 33.65 [29.26-38.35] |
| **Age group (n = 416)** |  |  |  |  |
| ≤29 | 17 (4.09) | 52.94 [30.21-74.52] | 5.88 [0.82-32.16] | 5.88 [0.82-32.16] |
| 30-39 | 50 (12.02) | 24.00 [14.14-37.71] | 22.00 [12.60-35.56] | 22.00 [12.60-35.56] |
| 40-49 | 142 (34.13) | 23.24 [17.00-30.91] | 28.87 [22.00-36.88] | 28.17 [21.37-36.14] |
| 50-59 | 135 (32.45) | 20.74 [14.71-28.43] | 48.15 [39.83-56.57] | 40.00 [32.06-48.50] |
| 60+ | 72 (17.31) | 12.50 [6.62-22.35] | 45.83 [34.71-57.39] | 47.22 [36.00-58.73] |
| Mean±SD | 49.32±10.43 | 46.25±10.02 | 52.30±8.65 | 52.33±9.02 |
| **Test** |  | 12.33** | 44.18*** | 34.15*** |
| **Sex (n=416)** |  |  |  |  |
| Male | 87 (20.91) | 11.49 [6.29-20.09] | 39.08 [29.40-49.70] | 6.90 [3.12-14.54] |
| Female | 329 (79.09) | 24.62 [20.25-29.58] | 35.56 [30.56-40.90] | 40.73 [35.53-46.14] |
| **Test** |  | 9.94*** | 0.36 | 77.77*** |
| **Marital status (n=416)** |  |  |  |  |
| Single | 62 (14.9) | 33.87 [23.21-46.47] | 22.58 [13.83-34.64] | 20.97 [12.56-32.88] |
| Married | 173 (41.59) | 15.61 [10.91-21.82] | 38.15 [31.20-45.62] | 26.59 [20.52-33.69] |
| D/S | 90 (21.63) | 28.89 [20.46-39.09] | 40.00 [30.40-50.44] | 41.11 [31.42-51.54] |
| Widow/Widower | 91 (21.88) | 18.68 [11.93-28.05] | 38.46 [29.04-48.84] | 48.35 [38.27-58.57] |
| **Test** |  | 11.37*** | 7.53* | 19.97*** |
| **Asset Quintile (n=416)** |  |  |  |  |
| Low | 145 (34.86) | 26.90 [20.29-34.71] | 37.93 [30.39-46.11] | 37.24 [29.74-45.41] |
| Middle | 162 (38.94) | 17.28 [12.20-23.92] | 32.10 [25.35-39.69] | 34.57 [27.63-42.23] |
| High | 109 (26.2) | 22.02 [15.20-30.78] | 40.37 [31.56-49.84] | 27.52 [19.94-36.66] |
| **Test** |  | 4.12 | 2.22 | 2.90 |
| **Educational level (n=416)** |  |  |  |  |
| None | 73 (17.55) | 19.18 [11.68-29.86] | 35.62 [25.49-47.21] | 35.62 [25.49-47.21] |
| Primary/JHS | 202 (48.56) | 23.76 [18.38-30.14] | 40.59 [34.02-47.52] | 37.62 [31.19-44.52] |
| SHS | 106 (25.48) | 19.81 [13.27-28.51] | 29.25 [21.36-38.61] | 30.19 [22.20-39.59] |
| Tertiary | 35 (8.41) | 22.86 [11.84-39.53] | 34.29 [20.58-51.23] | 17.14 [7.89-33.32] |
| **Test** |  | 1.04 | 4.16 | 8.64* |
| **Religion (n=416)** |  |  |  |  |
| Christian | 367 (88.22) | 21.80 [17.86-26.33] | 37.06 [32.25-42.13] | 33.24 [28.60-38.24] |
| Islam | 49 (11.78) | 22.45 [12.87-36.20] | 30.61 [19.35-44.79] | 36.73 [24.49-50.97] |
| **Test** |  | 0.01 | 0.84 | 0.23 |
| **Employment status (n=416)** |  |  |  |  |
| Not working | 29 (6.97) | 27.59 [14.41-46.30] | 48.28 [31.01-65.96] | 41.38 [25.17-59.70] |
| Employed | 88 (21.15) | 18.18 [11.43-27.67] | 29.55 [20.94-39.90] | 26.14 [18.01-36.31] |
| Retired | 24 (5.77) | 12.50 [4.07-32.46] | 50.00 [30.95-69.05] | 20.83 [8.92-41.41] |
| **BMI (n=400)** |  |  |  |  |
| Underweight | 18 (4.50) | 50.00 [28.36-71.64] | 5.56 [0.77-30.78] | 27.78 [12.03-51.97] |
| Normal | 145 (36.25) | 18.62 [13.08-25.82] | 35.17 [27.82-43.30] | 31.72 [24.65-39.75] |
| Overweight | 130 (32.50) | 17.69 [12.03-25.25] | 34.62 [26.92-43.20] | 36.92 [29.05-45.56] |
| Obese | 107 (26.75) | 23.36 [16.29-32.33] | 43.93 [34.82-53.46] | 33.64 [25.32-43.12] |
| **Test** |  | 7.79* | 31.19*** | 1.16 |

**NOTE**: **Abbreviation**; n=total number of samples involved as related to the specific variable; BMI=Body Mass Index. **Test** indicates analysis assessing differences in proportion after estimation.

**P-value Notation**: *p<0.05, ***p≤0.001

**Factors associated with depression, hypertension, and nephrotoxicity among Persons Living with HIV**

**Appendix Table 3: Sociodemographic factors associated with depression, hypertension, and nephrotoxicity among Persons Living with HIV**

| **Variable** | **Depression** | **Hypertension** | **Nephrotoxicity** |
| --- | --- | --- | --- |
|  | **OR [95%CI]** | **OR [95%CI]** | **OR [95%CI]** |
| **Age group** |  |  |  |
| ≤29 | 1 | 1 | 1 |
| 30-39 | 0.28 [0.09-0.89]* | 4.51 [0.54-38.00] | 4.51 [0.54-38.00] |
| 40-49 | 0.27 [0.10-0.75]* | 6.49 [0.83-50.71] | 6.27 [0.80-49.01] |
| 50-59 | 0.23 [0.08-0.66]** | 14.86 [1.91-115.5]** | 10.67 [1.37-83.0]* |
| 60+ | 0.13 [0.04-0.41]*** | 13.54 [1.69-107.8]* | 14.67 [1.37-83.0]* |
| **Sex** |  |  |  |
| Male | **1** | **1** | **1** |
| Female | 2.51 [1.24-5.09]** | 0.86 [0.53-1.40] | 9.28 [3.92-21.90]*** |
| **Marital status** |  |  |  |
| Married | **1** | **1** | **1** |
| Single | 2.77 [1.42-5.40]** | 0.47 [0.24-0.92]* | 0.73 [0.36-1.47] |
| D/S | 2.20 [1.19-4.06]* | 1.08 [0.64-1.82] | 1.93 [1.12-3.30]* |
| Widow/Widower | 1.24 [0.64-2.42] | 1.01 [0.60-1.71] | 2.58 [1.52-4.40]*** |
| **Asset Quintile** |  |  |  |
| Low | **1** | **1** | **1** |
| Middle | 0.57 [0.33-0.98]* | 0.77 [0.48-1.24] | 0.89 [0.56-1.42] |
| High | 0.77 [0.43-1.37] | 1.11 [0.66-1.84] | 0.64 [0.37-1.09] |
| **Educational level** |  |  |  |
| None | **1** | **1** | **1** |
| Primary/JHS | 1.31 [0.67-2.56] | 1.23 [0.71-2.15] | 1.09 [0.62-1.90] |
| SHS | 1.04 [0.49-2.21] | 0.75 [0.39-1.41] | 0.78 [0.41-1.47] |
| Tertiary | 1.25 [0.47-3.33] | 0.94 [0.40-2.20] | 0.37 [0.14-1.02] |
| **Religion** |  |  |  |
| Christian | **1** | **1** | **1** |
| Islam | 1.04 [0.51-2.12] | 0.75 [0.39-1.43] | 1.17 [0.63-2.17] |
| **Employment status** |  |  |  |
| Employed | **1** | **1** | **1** |
| Not working | 1.71 [0.64-4.56] | 2.23 [0.94-5.27] | 1.99 [0.82-4.80] |
| Retired | 0.64 [0.17-2.42] | 2.38 [0.95-6.00] | 0.74 [0.25-2.22] |
| Self-employed | 1.36 [0.74-2.51] | 1.34 [0.80-2.26]] | 1.62 [0.94-2.76] |
| **Region of residence** |  |  |  |
| GT. Accra | **1** | **1** | **1** |
| Other | 1.04 [0.55-1.94] | 0.97 [0.56-1.68] | 2.04 [1.19-3.47]** |
| **Number of children** |  |  |  |
| 5+ | **1** | **1** | **1** |
| None | 1.65 [0.63-4.33] | 0.33 [0.13-0.84]* | 0.49 [0.21-1.16] |
| 1-2 | 1.33 [0.59-2.97] | 0.77 [0.41-1.48] | 0.68 [0.35-1.29] |
| 3-4 | 1.10 [0.48-2.53] | 1.07 [0.55-2.08] | 0.65 [0.33-1.26] |
| **Has valid NHIS** |  |  |  |
| Yes | **1** | **1** | **1** |
| No | 1.26 [0.70-2.29] | 1.26 [0.78-2.12] | 0.57 [0.31-1.02] |

*Continued*

**Appendix Table 3 cont.: Sociodemographic factors associated with depression, hypertension, and nephrotoxicity among Persons Living with HIV**

| **Variable** | **Depression** | **Hypertension** | **Nephrotoxicity** |
| --- | --- | --- | --- |
|  | **OR [95%CI]** | **OR [95%CI]** | **OR [95%CI]** |
| **Social support** |  |  |  |
| Yes | **1** | **1** | **1** |
| No | 0.84 [0.49-1.43] | 1.06 [0.66-1.69] | 0.64 [0.40-1.02] |
| **Has sexual partner** |  |  |  |
| Yes | **1** | **1** | **1** |
| No | 1.44 [0.89-2.31] | 1.27 [0.84-1.89] | 2.30 [1.50-3.52]*** |
| **Use herbal medicine** |  |  |  |
| Yes | **1** | **1** | **1** |
| No | 0.79 [0.32-1.92] | 0.97 [0.43-2.17] | 1.22 [0.52-2.86] |
| **Co-morbid condition** |  |  |  |
| None |  |  |  |
| One only | 0.71 [0.40-1.25] | 5.16 [3.22-8.29]*** | 1.31 [0.82-2.08] |
| Two | 0.94 [0.19-4.63] | 2.27 [0.59-8.71] | 4.38 [1.07-17.93]* |

**NOTE**: **Abbreviation**; PR=Prevalence Ratio from Poisson regression; OR=Odd Ratio from Logistic regression; D/S=Divorce./Separated; JHS=Junior High School; SHS=Senior High School; GT. Accra= Greater Accra; NHIS=National Health Insurance Scheme; 1=1erence category used for inferences.

**P-value Notation**: *p<0.05; **p≤0.01; ***p≤0.001

**Appendix Table 4: HIV-related factors associated with depression, hypertension, and nephrotoxicity among Persons Living with HIV**

| **Variable** | **Depression** | **Hypertension** | **Nephrotoxicity** |
| --- | --- | --- | --- |
|  | **OR [95%CI]** | **OR [95%CI]** | **OR [95%CI]** |
| **Years of illness** |  |  |  |
| ≤1 |  |  |  |
| 2-5 | 0.98 [0.95-1.02] | 1.29 [0.50-3.34] | 1.26 [0.47-3.40] |
| 6-9 | 0.56 [0.23-1.36] | 1.38 [0.56-3.43] | 2.07 [0.82-5.21] |
| 10+ | 0.48 [0.23-1.04] | 1.95 [0.88-4.34] | 1.86 [0.81-4.28] |
| **Current ART combination** |  |  |  |
| TDF+DTG+3TC | **1** | **1** | **1** |
| ABC+DTG+3TC | 0.83 [0.23-2.98] | 1.09 [0.39-3.08] | 1.93 [0.71-5.29] |
| TDF+EFV+3TC | 1.35 [0.35-5.20] | 0.68 [0.18-2.62] | 1.11 [0.31-3.85] |
| Other | 1.05 [0.43-2.52] | 1.70 [0.81-3.56] | 0.46 [0.18-1.16] |
| **Years of current ART** |  |  |  |
| 3+ | **1** | **1** | **1** |
| ≤1 | 1.20 [0.49-2.96] | 0.60 [0.26-1.35] | 0.74 [0.30-1.82] |
| <2 | 0.71 [0.31-1.58] | 0.72 [0.36-1.44] | 1.56 [0.72-3.35] |
| **Total years on ART** |  |  |  |
| 11+ | **1** | **1** | **1** |
| ≤1 | 2.48 [1.10-5.58]* | 0.44 [0.18-1.06] | 0.61 [0.26-1.43] |
| 2-10 | 1.47 [0.91-2.43] | 0.78 [0.51-1.20] | 0.81 [0.53-1.25] |
| **Any past ART** |  |  |  |
| Yes | **1** | **1** | **1** |
| No | 2.10 [1.11-3.99]* | 0.60 [0.31-1.17] | 0.61 [0.30-1.20] |
| **Currently taking Septrin** |  |  |  |
| Yes | **1** | **1** | **1** |
| No | 0.53 [0.24-1.18] | 0.98 [0.45-2.13] | 0.64 [0.30-1.36] |

**Continued**

**Appendix Table 4 cont.: HIV-related factors associated with depression, hypertension, and nephrotoxicity among Persons Living with HIV**

| **Variable** | **Depression** | **Hypertension** | **Nephrotoxicity** |
| --- | --- | --- | --- |
|  | **OR [95%CI]** | **OR [95%CI]** | **OR [95%CI]** |
| **Disclosure of HIV** |  |  |  |
| None | **1** | **1** | **1** |
| Family member | 0.78 [0.37-1.63] | 1.11 [0.57-2.18] | 0.79 [0041-1.52] |
| Partner | 0.63 [0.28-1.37] | 0.94 [0.46-1.91] | 0.42 [0.21-0.86]* |
| Freinds+Others | 0.70 [0.21-2.30] | 0.46 [0.14-1.47] | 0.66 [0.23-1.86] |

**NOTE**: Abbreviation; PR=Prevalence Ratio from Poisson regression; OR=Odd Ratio from Logistic regression; ART= Antiretroviral Therapy; TDF=Tenofovir; DTG=Dolutegravir; 3TC=Lamivudine; ABC=Abacavir; EFV=Efavirenz; PR=Prevalence Ration; 1=Reference category used for inferences.

**P-value Notation**: *p<0.05

**Appendix Table 5: Sensitivity analysis showing anthropometric and nutritional indicators associated with depression, hypertension, and nephrotoxicity among Persons Living with HIV**

| **Variable** | **Depression** | **Hypertension** | **Nephrotoxicity** |
| --- | --- | --- | --- |
|  | **OR[95%CI]** | **OR[95%CI]** | **OR[95%CI]** |
| **BMI** |  |  |  |
| Normal | **1** | **1** | **1** |
| Underweight | 4.37 [1.58-12.06]* | 0.11 [0.01-0.84]* | 0.82 [0.27-2.46] |
| Overweight | 0.94 [0.51-1.74] | 0.97 [0.59-1.60] | 1.26 [0.76-2.07] |
| Obese | 1.33 [0.72-2.46] | 1.44 [0.86-2.41] | 1.09 [0.64-1.85] |
| **Fruit intake quintile** |  |  |  |
| High | **1** | **1** | **1** |
| Low | 1.90 [1.06-3.40]* | 1.31 [0.80-2.13] | 0.96 [0.58-1.57] |
| Middle | 1.26 [0.69-2.31] | 1.03 [0.63-1.69] | 0.74 [0.45-1.23] |
| **Vegetable intake quintile** |  |  |  |
| High | **1** | **1** | **1** |
| Low | 1.87 [1.05-3.32] | 0.88 [0.53-1.45] | 0.70 [0.43-1.16] |
| Middle | 1.18 [0.65-2.17] | 1.38 [0.53-1.45] | 0.69 [0.42-1.13] |
| **Add salt at table** |  |  |  |
| Never | **1** | **1** | **1** |
| Sometimes | 1.92 [1.14-3.25]* | 0.76 [0.46-1.25] | 0.58 [0.34-0.99]* |
| **Food insecurity** |  |  |  |
| No | **1** | **1** | **1** |
| Yes | 1.53 [0.93-2.53] | 1.67 [1.07-2.59]* | 0.99 [0.63-1.58] |

**NOTE**: **Abbreviation**; PR=Prevalence Ratio from Poisson regression; OR=Odd Ratio from Logistic regression; 1=Reference category used for inferences; BMI=Body Mass Index.

**P-value Notation**: *p<0.05; **p≤0.01

Appendix Table 6: Distribution of depression, hypertension and nephrotoxicity by sex and age groups

| **Sex** | **Outcome** | **Age group** | | | | **P-value** |
| --- | --- | --- | --- | --- | --- | --- |
|  |  | **≤39** | **40-49** | **50-59** | **60+** |  |
|  |  | n(%) | n(%) | n(%) | n(%) |  |
| Male | Depression | 1(5.9) | 1(7.7) | 6(17.1) | 2(9.1) | 0.593 |
|  | Hypertension | 2(11.8) | 4(30.8) | 21(60.0) | 7(31.8) | 0.006 |
|  | Nephrotoxicity | 1(5.9) | 0(0.0) | 2(5.7) | 3(13.6) | 0.451 |
|  |  |  |  |  |  |  |
| Female | Depression | 20(40.0) | 32(24.8) | 22(22.0) | 7(14.0) | 0.020 |
|  | Hypertension | 10(20.0) | 37(28.7) | 44(44.0) | 26(52.0) | <0.001 |
|  | Nephrotoxicity | 11(22.0) | 40(31.0) | 52(52.0) | 31(62.0) | <0.001 |
